# Health Behaviors, Knowledge, Life Satisfaction, and Wellbeing in People with Mental Illness across Four Countries and Comparisons with Normative Sample

**DOI:** 10.3389/fpsyt.2016.00145

**Published:** 2016-08-22

**Authors:** Natalie Parletta, Yousef Aljeesh, Bernhard T. Baune

**Affiliations:** ^1^Centre for Population Health Research, School of Health Sciences, University of South Australia, Adelaide, SA, Australia; ^2^Faculty of Nursing, Islamic University of Gaza, Gaza, Palestine; ^3^Department of Psychiatry, University of Adelaide, Adelaide, SA, Australia

**Keywords:** mental illness, diet, lifestyle, sleep, health knowledge, education

## Abstract

**Background:**

People with chronic mental illness have poorer physical health and higher mortality than the general population. We investigated lifestyle factors in people with mental illness across four countries and compared with a normative sample.

**Design and methods:**

Data were collected from *N* = 672 people (Germany, *n* = 375; Palestine, *n* = 192; London, *n* = 63; Australia, *n* = 42) with substance abuse disorder (*n* = 224), schizophrenia (*n* = 158), mood disorders (*n* = 227), and somatoform disorders (*n* = 63). The General Health Behaviour Questionnaire measured behaviors and knowledge related to nutrition, physical activity, alcohol, smoking, sleep, life satisfaction, and wellbeing. The normative samples were derived from a German population (*N* = 1,019). Data were analyzed using ANOVAs and *t*-tests.

**Results:**

The Palestine sample did not differ from the Western samples on reported life satisfaction and wellbeing. However, they reported unhealthier diets, less physical activity, and lower knowledge about the impact of diet, physical activity, smoking, and sleep on health than the Western samples. Comparing the Western and normative samples, people with mental illness reported lower intake of healthy foods/drinks, higher intake of unhealthy foods, higher exercise, higher alcohol consumption, less cigarettes, less sleep, and more sleep problems. Their knowledge was lower for nutrition, physical activity, and smoking. All participants reported lower life satisfaction and wellbeing than the normative sample (*P*-values <0.001).

**Conclusion:**

Education on health-related lifestyle factors present important targets for primary care, quality of life and prevention of illness in people with mental illness. Further research will clarify specific predictors of health behaviors in each country.

## Introduction

A 2007 global survey identified that the estimated lifetime prevalence of having one or more mental disorders ranged from 47.4% in the United States to 12% in Nigeria (1), with more than one-third of respondents from five countries reporting symptoms of lifetime mental illness, more than a quarter in six countries and more than one-sixth in four countries. The authors concluded that mental disorders are common. Given the enormous societal and personal burdens of mental disorders and their lifetime prevalence, research, and public health interventions focused on early detection and treatment are needed (1).

Not only does chronic mental illness lead to substantially reduced quality of life, social, and economic burden but it is also compounded by high rates of physical illness. People with mental illness are more likely to suffer from a range of physical health problems, particularly metabolic syndrome, diabetes, and cardiovascular diseases (CVD) (2–5), contributing to a 20-year reduced life expectancy in this vulnerable population (6). Although anti-psychotic medications contribute to this risk (7), lifestyle factors, such as higher intake of energy-dense, highly processed food (8), lower physical activity levels (9), and higher smoking rates (10), have also been reported.

Given that lifestyle factors, such as smoking, diet, and physical activity have been identified as risk factors for both physical and mental illness, they may contribute to common underlying biological mechanisms for both conditions (11). Supporting evidence comes from a meta-analysis of epidemiological studies showing a significant association between depression and metabolic syndrome (12), a cluster of risk factors for CVD, including hyperglycemia and/or insulin resistance, hypertension, abdominal adiposity, and hyperlipidemia. Prospective studies identified in the latter review showed that the association is bidirectional – i.e., depression was predictive of metabolic syndrome and conversely metabolic syndrome predicted depression. Data from the Canadian Mental Health Association indicated that 2.8% of people with no chronic physical condition had a mood disorder compared to 9.3–11.4% of people with chronic conditions, such as diabetes, cancer, and heart disease and a comorbid mood disorder (13). A review of studies in patients with mood disorders found that between 25 and 67% were obese. Conversely, 32% of patients with obesity had a lifetime mood disorder across a range of studies, and when this was restricted to patients who had been diagnosed using a structured clinical interview the rate was 41% (14). A meta-analysis of longitudinal studies identified that depressed adults have a 37% increased risk of developing diabetes (15), further suggestive of common underlying mechanisms and, hence, similar risk factors.

However, relatively little focus has been given to physical health in people with mental illness and lifestyle factors that have the potential to prevent and reduce the significant social, economic, and personal burden of poor physical health and quality of life in mental illness globally. This study aimed to investigate lifestyle factors in people with mental illness from four countries and compare with a normative sample. Specifically, the study assessed self-reported dietary patterns, physical activity, alcohol consumption, smoking, sleep patterns, knowledge of the impact of these lifestyle behaviors on health, and overall life satisfaction and perceived wellbeing. It was hypothesized that people with mental illness would have poorer health behaviors, lower knowledge about the impact of these behaviors on health, and lower self-reported life satisfaction and wellbeing.

## Materials and Methods

### Participants

Participants were community-dwelling patients with mental illness recruited from outpatient clinics across four countries from 2001 to 2008 for the purpose of this study. The samples from the respective countries come from single centers with consistent admission criteria per center. The sampling strategy was to draw on patients who were admitted to hospital or were outpatients for the treatment of their mental illness. All centers were tertiary referral centers at University Hospitals, hence, they included patients with a larger degree of symptom severity and the patients had recurrent episodes of their illness. This was consistent across all countries and centers. The inclusion criteria into the study were willingness to participate, adult age group, and referral to the center for treatment of their mental illness. Patients not participating were those with inability to fill out the questionnaire, cognitive impairment, acute physical illness compromising the ability to take part in the study, or language barrier. Every country had included one such center. Six hundred and ninety-seven patients gave informed consent to participate on intake into the clinics, with a response rate of 76.2% across all locations: Germany (*n* = 387), Palestine (*n* = 200), London (*n* = 67), and Australia (*n* = 43). International Classification of Diseases (ICD)-10 F categories were used to classify patients into seven diagnostic groups: F1 organic (*n* = 2), substance abuse (*n* = 224), F2 schizophrenia and schizoaffective (*n* = 158), F3 depressive (*n* = 227), F4 neurotic and somatoform (*n* = 63), F5 physiological (*n* = 2), and F6 personality disorders (*n* = 21). Those with organic, physiologically related, and personality disorders were excluded, leaving a sample of *N* = 672. Ethics approval was provided by the ethics committee at the University of Bielefeld, Germany, and relevant local ethics committees, and the study was performed in accordance with the ethical standards laid down in the 1964 Declaration of Helsinki and its later amendments.

### Measures

Patients completed a questionnaire on intake into the outpatient clinics. Demographic information requested included gender, age, marital and family status, living situation, occupation, and education (from 1 = “no secondary school qualification” to 6 = “university degree”). Clinical staff measured weight and height, from which BMI (kg/m^2^) was calculated. The German General Health Behaviour Questionnaire (GHBQ) was used to assess lifestyle behaviors, knowledge, and life satisfaction as it has shown acceptable validity and reliability in samples with mental illness (16). The questionnaire was translated into Arabic and English by two independent translators and results were checked for inconsistencies. Face and content validity were checked by submitting the questionnaire to experts in the field in the respective language for back and forward translation. Dependent variables were calculated according to instructions in the GHBQ manual.

Frequency of eating nominated foods, undertaking various physical activities, and consumption of different types of alcohol were measured on 4-point Likert scales (1 = never; 4 = daily) and mean values were obtained. Food was reported in the manual as two separate categories of frequency of healthy foods and unhealthy foods consumed based on principal components analysis. Frequency of smoking was measured by number of cigarettes smoked per day and for how many years. Sleep was measured by asking about number of hours slept per night, difficulties with staying asleep, and waking up during the night. Knowledge regarding the impact of lifestyle behaviors on health was derived by asking participants to circle how many diseases are associated with each behavior, which were then summed. General wellbeing was measured by asking how satisfied the respondent is with their life in general and to assess their present wellbeing (each on a scale of −3 = extremely unsatisfied to +3 = extremely satisfied). Further questions asked about different areas of life that contribute to satisfaction and wellbeing, and those that contribute to problems or difficulties (both ranging from 1 = not at all to 5 = very true). These were summed to provide a score of the total areas of life perceived to contribute to satisfaction and wellbeing or problems and difficulties.

Normative data are provided by the GBHQ manual (16). These normative data comprise healthy adult individuals derived from the general population in Germany (*N* = 1,019). The mean age was 51.1 years (SD 13.1, range 18–83). The gender distribution of respondents was 63.8% female and 36.2% male.

### Statistical Analysis

One sample *t*-tests were used to compare the mental illness samples with the normative sample. Differences between the Western countries and the Middle Eastern sample from Palestine were previously reported for nutritional habits (17). Therefore, before pooling data from the four countries together for comparisons with the normative sample, differences between the Western countries were checked for all variables using one-way ANOVAs and between the Western and Middle Eastern samples using independent *t*-tests. On variables where a country differed to the other countries, comparative analyses with the normative sample were done without that sample to avoid confounding.

## Results

Participants were aged 15–78 years (M = 39.80, SD = 11.45), consisting of 56.7% males and 43.3% females. Overall, half of participants either had no secondary school qualification or had only completed comprehensive school, around 30% had completed secondary school, and 11% had completed a university degree. Participants in all countries were, on average, overweight, and in Australia the average BMI was in the obese range. Demographic information, broken down by country, is presented in Table 1.

**Table 1:** **Demographic data for sample broken down by country**.

	Germany (*n* = 375)	UK, London (*n* = 63)	Australia (*n* = 42)	Palestine (*n* = 192)
Age, M (SD)	41.42 (10.81)	39.44 (11.45)	50.74 (12.71)	34.39 (9.65)
Gender	59% male	52.4% male	32.6% male	57.5% male
*Education level*				
No secondary school	5.1%	4.8%	16.7%	56.4%
Comprehensive school	43.1%	8.1%	4.8%	0.6%
Secondary school	33.6%	22.6%	48.3%	18.2%
University degree	8.1%	30.6%	4.8%	9.9%
*Marital status*				
Married	33.5%	17.5%	30.2%	50.3%
Partnered	7%	9.5%	11.6%	0.5%
Single	32%	61.9%	23.3%	33.7%
Widowed	4.6%	–	9.3%	1%
Separated	21.7%	9.5%	23.3%	14.5%
No. children, M (SD)	1.08 (1.13)	0.52 (1.11)	2.07 (1.75)	2.87 (3.07)
BMI (kg/m^2^)	25.00 (4.81)	24.94 (4.48)	31.43 (7.77)	27.36 (7.74)
*ICD-10 Category (n)*				
F1: mental and behavioral disorders due to psychoactive substance abuse	221	–	–	3
F2: schizophrenia, schizotypal, and delusional disorders	25	22	–	111
F3: mood (affective) disorders	92	31	43	61
F4: neurotic, stress-related, and somatoform disorders	35	10	–	18
*Occupation (n)*				
Professional	14	6	6	4
Self-employed	13	4	3	7
Salaried manager	32	1	1	–
Government official	13	10	2	14
Farmer	5	–	–	5
Laborer	120	–	4	30
Homemaker	30	4	4	56
Student	11	7	1	14
Pensioner/retired	27	10	16	6
Unemployed	100	18	5	48

One-way ANOVAs revealed no significant differences on outcome variables between the Western countries. Comparison of Western and Palestinian samples showed that they were significantly different on all dependent variables reported apart from overall life satisfaction and wellbeing, and number of areas of life contributing to difficulties or problems (Table 2). Specifically, people from the Palestinian sample reported higher consumption of both healthy and unhealthy food and drinks, less physical activity, less cigarettes, more sleep, and less sleep problems. They had lower knowledge of the impact of diet, exercise, smoking, and sleep on health. Because of these differences, comparisons with the normative sample were done with the Western samples for all variables apart from those that did not differ between countries (Table 3).

**Table 2:** **Comparison of Western and Palestinian samples**.

Variable	Region	*N*	M (SD)	*P* for difference
Consumption of healthy food and drinks	Western	479	2.48 (0.41)	<0.001
	Palestine	182	2.65 (0.38)	
Consumption of traditional (unhealthy) food	Western	479	2.50 (0.32)	<0.001
	Palestine	179	2.74 (0.29)	
Lay assessment relating to diet and health knowledge	Western	478	57.18 (21.69)	<0.001
	Palestine	193	35.85 (14.73)	
Extent of physical activity	Western	497	1.88 (0.37)	<0.001
	Palestine	189	1.75 (0.33)	
Lay assessment relating to exercise and health knowledge	Western	497	71.89 (13.87)	<0.001
	Palestine	193	61.72 (14.60)	
Frequency of consumed alcohol	Western	442	2.07 (0.70)	<0.001
	Palestine	147	1.00 (0.00)	
Lay assessment relating to alcohol and health knowledge	Western	470	56.70 (18.60)	
	Palestine	193	–	
Number of cigarettes/tobacco smoked per day	Western	475	15.56 (14.40)	<0.001
	Palestine	186	9.20 (14.32)	
Lay assessment relating to smoking and health knowledge	Western	471	77.26 (13.90)	<0.001
	Palestine	193	68.65 (8.79)	
No. of hours sleep per night	Western	478	6.80 (1.81)	<0.001
	Palestine	193	7.26 (2.51)	
Sleep problems	Western	467	3.23 (1.07)	0.004
	Palestine	163	2.94 (1.10)	
Negative mood after getting up	Western	478	3.00 (0.59)	<0.001
	Palestine	181	2.81 (0.68)	
Lay assessment relating to sleep and health knowledge	Western	476	79.66 (11.18)	<0.001
	Palestine	193	75.23 (8.54)	
Life satisfaction	Western	475	−0.28 (1.89)	0.258
	Palestine	193	−0.48 (2.16)	
Current wellbeing	Western	474	−0.19 (1.90)	0.760
	Palestine	193	−0.24 (1.76)	
Areas of life contributing to wellbeing and life satisfaction	Western	446	3.27 (1.01)	<0.001
	Palestine	191	2.45 (0.76)	
Areas of life contributing to difficulties and problems	Western	437	2.61 (0.84)	0.726
	Palestine	190	2.64 (1.16)	
Total dissatisfaction with problems and impact on health	Western	452	−1.04 (1.51)	<0.001
	Palestine	193	0.09 (1.60)	

**Table 3:** **Comparison of samples with mental illness (all countries *N* = 672; Western sample *n* = 479) and normative sample (*N* = 1,019)**.

Variable	Region	M (SD)	*P* for difference
Consumption of healthy food and drinks	Western	2.48 (0.41)	<0.001
	Normative	2.84 (0.41)	
Consumption of traditional (unhealthy) food	Western	2.50 (0.32)	<0.001
	Normative	2.41 (0.33)	
Lay assessment relating to diet and health knowledge	Western	57.18 (21.69)	<0.001
	Normative	68.00 (21.35)	
Extent of exercise	Western	1.88 (0.37)	<0.001
	Normative	1.82 (0.37)	
Lay assessment relating to exercise and health knowledge	Western	71.89 (13.87)	<0.001
	Normative	75.96 (19.37)	
Frequency of consumed alcohol	Western	2.07 (0.70)	<0.001
	Normative	1.85 (0.38)	
Lay assessment relating to alcohol and health knowledge	Western	55.06 (17.93)	<0.001
	Normative	62.19 (13.60)	
Number of cigarettes/tobacco smoked per day	Western	15.56 (14.40)	0.001
	Normative	17.70 (10.60)	
Lay assessment relating to smoking and health knowledge	Western	77.26 (13.90)	0.455
	Normative	77.74 (13.92)	
No. of hours sleep per night	Western	6.80 (1.81)	<0.001
	Normative	7.23 (0.94)	
Sleep problems	Western	3.23 (1.07)	<0.001
	Normative	2.68 (1.07)	
Negative mood after getting up	Western	3.00 (0.59)	<0.001
	Normative	2.79 (0.89)	
Total contentment with sleep and perceived impact on health	Western	−0.08 (1.86)	<0.001
	Normative	0.94 (1.84)	
Lay assessment relating to sleep and health knowledge	Western	79.66 (11.18)	<0.001
	Normative	77.35 (13.95)	
Life satisfaction	All countries	−0.34 (1.97)	<0.001
	Normative	1.58 (1.40)	
Current wellbeing	All countries	−0.20 (1.86)	<0.001
	Normative	1.14 (1.66)	
Areas of life contributing to difficulties and problems	All countries	2.62 (0.95)	<0.001
	Normative	2.23 (0.83)	
Areas of life contributing to wellbeing and life satisfaction	Western	3.27 (1.01)	<0.001
	Normative	4.02 (0.68)	
Total dissatisfaction with problems and impact on health	Western	−1.04 (1.51)	<0.001
	Normative	0.50 (1.69)	

As shown in Table 3, people with mental illness from the Western countries reported significantly lower consumption of healthy food and drinks, higher consumption of “traditional” unhealthy food, higher levels of physical activity, higher alcohol consumption, less smoking, less sleep per night, higher reported sleep problems, negative mood after getting up and greater dissatisfaction with sleep, and perceived impact on their health than the normative sample. People with mental illness had significantly lower knowledge of the health risks associated with unhealthy diet, low physical activity levels, alcohol consumption, and smoking and higher identification of health risks associated with sleep problems. When smoking rates were broken down we observed that the most number of cigarettes smoked per day were by the German sample of people with mental illness (M = 17.92, SD = 14.39). When they were compared with the normative sample (from Germany), the difference was not significant (*P* = 0.773). People with mental illness from all countries reported significantly lower life satisfaction and perceived wellbeing than the normative sample. The Western sample reported less areas of life contributing to wellbeing and life satisfaction and greater dissatisfaction with their problems and perceived impact on their health. All samples reported more areas of life contributing to problems and difficulties than the normative sample.

## Discussion

This study aimed to compare lifestyle behaviors of people with mental illness from four different countries with a normative sample that was derived from Germany. The Middle Eastern sample differed significantly from the three Western samples on lifestyle and knowledge measures but not on life satisfaction and perceived wellbeing. Where they differed, comparisons were made between the Western samples with mental illness and the normative sample; otherwise, comparisons were done with all samples. People with mental illness from the Western countries reported less healthy food and drink and more “traditional” unhealthy food consumption; they reported higher alcohol consumption, less sleep per night and higher sleep problems plus negative mood after getting up than the normative sample. Unexpectedly, and contrary to other studies, the people with mental illness reported higher levels of physical activity and less smoking than the normative sample. However, it is important to note that the reported level of physical activity was low in all samples. The number of cigarettes smoked daily by the German mental illness sample was not higher than rates reported for the normative sample from Germany which was published in 1995. Given that the mental illness data were collected between 2001 and 2008, rates of smoking in the general population are likely to have reduced with widespread public health campaigns. People with mental illness from the Western countries had lower knowledge of the health risks associated with unhealthy diet, low exercise levels, alcohol consumption, and smoking. Their identification of health risks associated with sleep problems was higher than the normative sample. People with mental illness from all countries, including the Middle Eastern sample, reported notably lower life satisfaction and perceived wellbeing than the normative sample. They all reported more areas of life contributing to problems and difficulties and the Western samples reported less areas of life contributing to wellbeing and life satisfaction. Compared to the Western samples, the Middle Eastern sample reported higher intake of healthy food, less smoking and less sleep problems, and reported even higher intake of unhealthy food, lower physical activity, and lower knowledge of the impact of all lifestyle factors on health compared with the Western samples.

Diets higher in fat and lower in fiber in people with schizophrenia have been identified, as well as smoking and low physical activity, and were found independently of anti-psychotic medication and socio-economic deprivation (18). Similar to our results, Osborn et al. (18) identified lower CVD knowledge in people with mental illness. Our findings further support the importance of health education in people with mental illness regarding the well-established lifestyle risk factors for CVD, including diet, physical activity, and smoking.

Education for people with mental illness needs to take into consideration that some have lower literacy levels, and may have short attentions spans and memory problems. Therefore, education programs need to have simple messages and would ideally be incorporated into rehabilitation programs with hands-on learning – e.g., interactive education workshops, cooking workshops, and physical activity education that is targeted at individual abilities and preferences. Sixteen interventions that focused on weight loss or weight gain prevention for community-dwelling people with mental illness were identified in a review (19). They were of varying duration and intensity, targeting healthy eating, exercise and fitness, healthy living, goal setting, and self-regulation skills. Overall the interventions were successful in reducing weight/weight gain, although this was not necessarily sustained. The program that included practical skills for healthy eating, shopping, and meal preparation was reported as most successful. We piloted cooking workshops teaching basic cooking skills, exposure to healthy whole foods, and nutrition education with people who have serious mental illness in Australia. These workshops were very popular and showed successful preliminary outcomes (20). A recent study reported that a nutrition intervention in youth with first-episode psychosis successfully improved their dietary behaviors, particularly reduced intake of discretionary foods high in fat and sugar, and increased vegetable intake that is associated with reduced CVD risk (21).

Vancampfort et al. (22) reviewed evidence for the role of physical activity in people with schizophrenia, and concluded that physical activity interventions in this population can improve metabolic health, physical fitness, health-related behavior, and mental health. A Cochrane review concluded that regular exercise programs are possible in this population, and can have positive mental and physical health benefits; however, more randomized controlled trials are required (23).

Our study supports other reports that people with mental illness have greater problems with sleep. Sleep problems are estimated to affect 50–80% of psychiatric patients compared with 10–18% of people from the general population (24). It is estimated that 20–40% of people with psychiatric disorders experience insomnia (25), and people with insomnia are reported to be nine times more likely to suffer from a major mood disorder (26). Furthermore, evidence shows that sleep problems have a detrimental impact on symptoms of psychiatric illness, further impair function, and persist even when symptoms are treated (25). Traditionally, sleep problems were thought to be a symptom of mental illness. However, sleep is now thought to be a possible contributor to psychiatric disorders in a complex bidirectional fashion (27). Interestingly, people with mental illness in our study showed a heightened awareness of the health risks associated with sleep problems compared to the normative sample. They also reported low satisfaction with sleep and its perceived impact on their health. These findings suggest a need for intervention, rather than education, to assist people with mental illness who have sleep problems.

A position statement released by the European Psychiatry Association called for a greater awareness by health professionals of cardiovascular morbidity in people with mental illness and screening for diabetes and CVD risk factors (28). Our data support this and other calls for lifestyle factors, such as diet/nutrition, physical activity, and sleep to also be addressed in treatment of people with mental illness (18, 29). Education of people with mental illness and high at risk groups can further empower them to take charge of their own health.

Quality of life is lower in people with mental illness than in the general population (30). This is compounded by comorbid physical illness; however, medical conditions are undertreated in clinical psychiatric practice (31). A recent Australian Health Survey identified that one in nine Australians have a comorbid mental and physical condition, and that these people suffer substantially greater psychological distress and profound disability than people without mental disorders or physical conditions (32). Across four countries, our sample of people with mental illness was, on average, overweight or obese, putting them at risk for comorbid physical illness and associated risk factors. Our sample similarly reported low levels of life satisfaction and wellbeing and a high level of dissatisfaction with their problems and the perceived impact on their health. This further highlights the significant global, personal, and social costs of chronic mental illness.

The results of this study are limited by its cross-sectional nature and normative data being restricted to a German sample. Furthermore, while the present data were collected between 2001 and 2008, data from the normative sample were collected in 1995 so some of the differences may be attributed to when the data were collected. The sample was a convenience sample so not necessarily representative, although respondents were all recruited from tertiary care centers and the response rate was good. The dietary component of the GHBQ was based on German foods that may have contributed to cross-cultural differences, although the majority of “healthy” versus “unhealthy” foods would be similar across countries. Finally, there are possible confounding variables and cross-cultural differences, particularly between the Western and Middle Eastern samples. Importantly, other studies have found that predictors of health outcomes differ between countries (33–36). We are currently analyzing a number of potential predictors of health behaviors in this sample across countries, including demographic variables, motivations, internal and external barriers, and health knowledge (37).

## Summary and Conclusion

People with mental illness have a high rate of chronic physical illnesses and these comorbidities are associated with more severe trajectory of the mental illness, reduced quality of life, and higher rate of premature mortality. We identified lifestyle risk factors for chronic illness in people from four countries with mental illness and a lack of knowledge regarding the impact of lifestyle factors on health. This presents an important target for education of people with mental illness, particularly regarding diet, physical activity, and smoking, which can help empower them to take charge of their own health. Our Western samples seemed to have an awareness of the impact that poor sleep had on them and expressed dissatisfaction about the impact of sleep problems on their health, suggesting that therapeutic approaches to sleep problems are warranted.

Although a complex array of influences will contribute to the high comorbidity between poor physical and mental health, such as environmental, genetic, and psycho-social factors, there may also be common biological mechanisms underpinning these comorbidities, such as inflammation (38–41). More research is needed to investigate the direct contribution that lifestyle risk factors may have on mental illness (42, 43), and targets for prevention via tailored public health campaigns and individualized advice for at-risk populations. Further analysis of data from this sample will identify specific predictors of health behaviors within each country that can be targeted (37).

## Author Contributions

NP analyzed the data and prepared the manuscript. YA participated in the design of the study and data collection. BB conceived of the study and its design, organized data collection, and provided input to statistical analysis. All authors read and approved the final manuscript.

## Conflict of Interest Statement

The authors declare that the research was conducted in the absence of any commercial or financial relationships that could be construed as a potential conflict of interest.
